# Greater mouse-tailed bats use their tail as a tactile sensor when navigating backwards

**DOI:** 10.1016/j.isci.2025.112014

**Published:** 2025-02-17

**Authors:** Sahar Hajyahia, Mor Taub, Ofri Eitan, Orit Dashevsky, Yossi Yovel

**Affiliations:** 1 School of Zoology, Faculty of Life Sciences, Tel Aviv University, Tel Aviv 6997801, Israel; 2 Sagol School of Neuroscience, Tel Aviv University, Tel Aviv 6997801, Israel

**Keywords:** Wildlife behavior, Biological sciences, Zoology

## Abstract

Animals use a wide arsenal of sensory modalities to orient, often combining information from different modalities to improve sensing. Animals mostly move forward and hence most of their sensory organs are frontal. In some situations, moving backwards is a necessity and some animals have evolved designated sensory strategies. The greater mouse-tailed bats *(Rhinopoma microphyllum)* belong to one of few bat families that possess a long free tail which they wag in a pendulum like pattern when moving backwards up walls and between obstacles. We show that greater mouse-tailed bats use their tail to navigate around obstacles and are hindered when their tail is anesthetized. Additionally, we find that they use their tail to discriminate between textures and can sense subtle changes. We suggest that the use of the tail as a tactile sensor enables these bats to move backwards quickly when other sensory modalities are useless.

## Highlights

• Greater mouse-tailed bats use their tail as a tactile sensory tool
• Bats with intact tails navigated around obstacles faster than with anesthetized tails
• Bats could discriminate between surface textures with the use of the tail

## Graphical abstract

**Figure:**
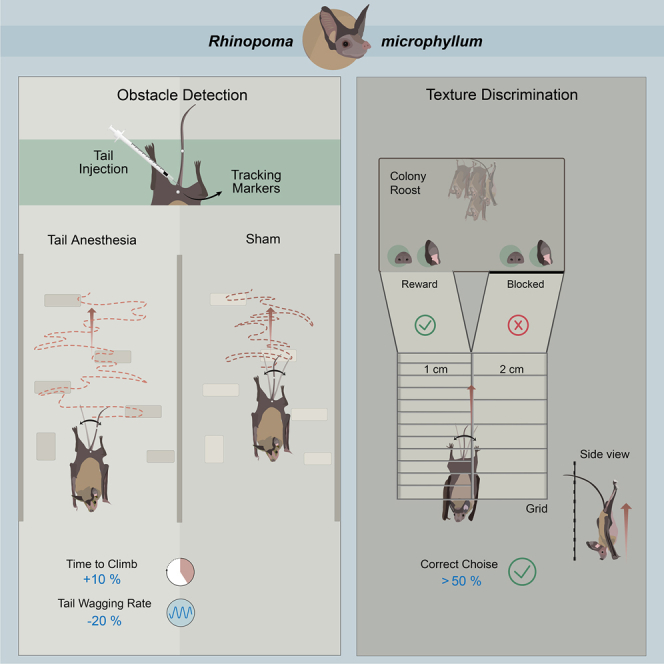

## Introduction

The mammalian tail exhibits a variety of morphological shapes and a diversity of mechanical and behavioral functions including movement,^1^^,^^2^^,^^3^ defense,^4^^,^^5^ and communication.^6^^,^^7^ The versatility of the tail’s function makes it an important organ for the survival of many animals.^8^ Cats, rodents, and some primates use their tails to balance themselves while walking on narrow surfaces or climbing trees.^3^^,^^9^ Ungulates use their tails to swat away flies,^4^ while rats use it to protect their head during an attack.^5^ The position of the tail can indicate the state of an animal while the shape and movement pattern can convey social information to other individuals.^9^ The tail can also be used as a sensory tool, for example, when moving backwards into a tunnel or while digging borrows.^10^^,^^11^

Generally speaking, bats’ tails have been mostly reduced during their evolution. Most bat species have short tails embedded within the tail membrane which acts as an additional surface area for the overall wing. It produces lift and reduces wing loading, improving maneuverability in flight,^12^^,^^13^^,^^14^ and it also assists insect capture.^15^^,^^16^^,^^17^ Variations in bat tail morphology have been shown to correlate with flight and foraging style.^18^^,^^19^ Fast flying bats have smaller tail membranes since tail membranes typically increase the mechanical cost of flight;^20^ however, the longer (membrane embedded) tail of *Myotis blythii* was shown to contribute to improved maneuverability over its sympatric species *Myotis myotis*.^21^ These variations in bat-tail membrane size are most likely the result of a trade-off between energetic costs, and aerodynamic or foraging benefits.^13^^,^^19^^,^^20^^,^^22^ When it comes to sensing, the wings and tail membrane of bats are lined with microscopic tactile hairs.^23^^,^^24^^,^^25^ Big brown bats (*Eptesicus fuscus*) were found to use these hairs for flight control and suffered diminished performance without them.^26^

The greater mouse-tailed bats *(Rhinopoma microphyllum)* are swift and maneuverable fliers,^14^^,^^27^ who forage for insects at relatively high altitudes and open spaces.^28^^,^^29^^,^^30^ Greater mouse-tailed bats have free, protruding, 41–63 mm long tails, uniquely long for a bat, with small interfemoral membranes—less than one-fourth of the tail’s length. Their fast flight and foraging style may explain their reduced uropatagium.^20^ They also have tactile hairs at the tip of their tails that have been hypothesized to be used for tactile sensing.^31^ While perching, these bats wag their long tails in a pendulum-like pattern when they are disturbed, or before taking off in flight^27^ as well as when they crawl backwards up in between obstacles, seemingly using the tail as blind people use a cane. Despite these observations, the sensory role of the tail has not yet been investigated, leaving the function of the tail-wagging behavior obscure.

In this study we aimed to investigate the role of greater mouse-tailed bats’ tail as a tactile active sensor. We hypothesized that these bats would use their tails to sense objects located behind them and adjust their movement accordingly. We predicted that reducing sensation in the tail (using anesthesia) would reduce obstacle avoidance efficiency and slow the bats’ movement. In addition, since they often move backwards in dark roosts, we hypothesized that the bats would be able to use sensory information from the tail to distinguish between different surface textures. We designed two behavioral experiments that demonstrate the use of the tail as a tactile sensory tool allowing the bats to move around obstacles and discriminate between textures in the environment. Our results show that *R. microphyllum* use their long tails as a tactile sensor to aid in navigation while moving backwards.

## Results

### Experiment 1: Obstacle avoidance

**Figure 1:**
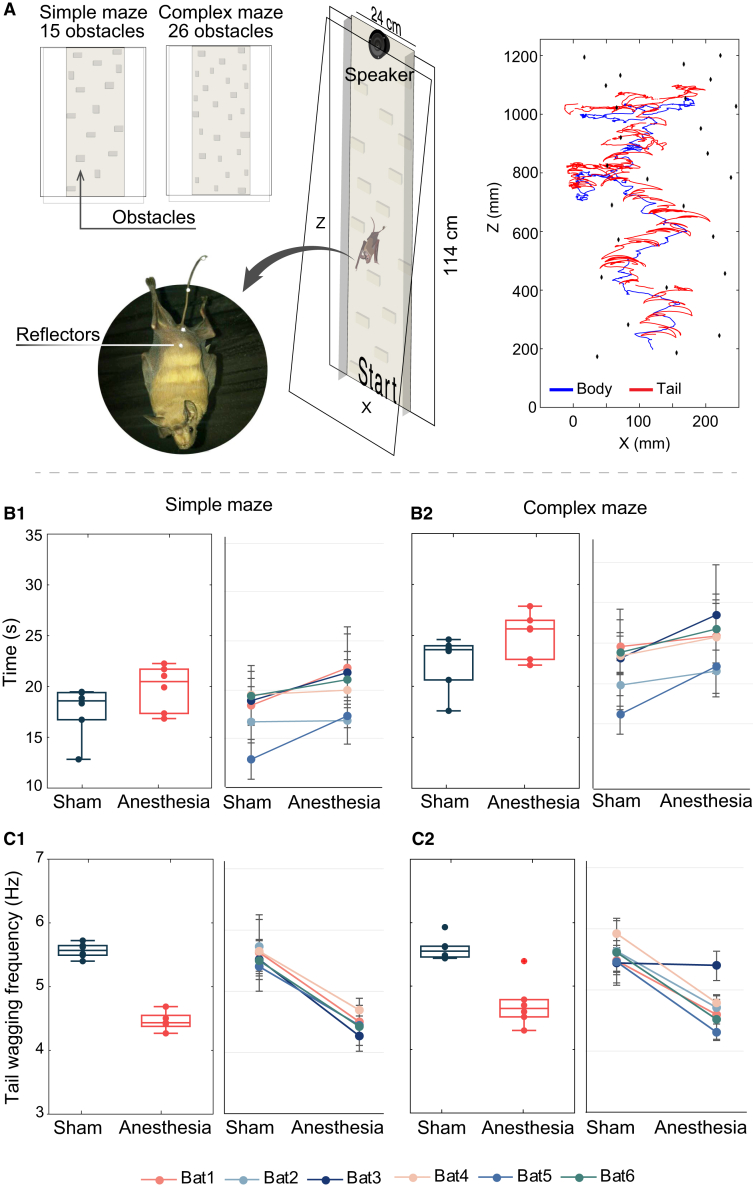
Bats use their tail as a tactile sensor for obstacle avoidance (A) Set-up of the obstacle maze experiment. Perspex glass obstacles were attached to a perpendicular Perspex board covered with felt to allow climbing. An additional transparent board covered the maze to prevent the bat from exiting the maze and allowed video recording and tracking of the experiment. A speaker placed at the top of the maze played social calls to attract the bats to the top. Two maze complexities were used in the experiment: a simple, 15 obstacle maze and a complex 26 obstacle maze. Two reflectors placed on the bat (middle of the tail and lower body) were used to track its movement. An example of the 2D tracking of the body (blue) and middle tail (red) reflectors during one trial is shown. (B) The time needed to reach the top of the simple (B1) and complex (B2) maze under the control (sham) and sensory block treatment (anesthesia). Results are shown for all bats (boxplots) and for individual bats – lines (mean ± SD). (C) Tail wagging frequency under the control (sham) and sensory block treatment (anesthesia) in the simple (C1) and complex (C2) maze. For all Boxplots, lines represent the median and lower and upper quartiles. Circles represent individual-trial data points for each condition (mean of each bat, *n* = 6 bats). The whiskers extend to the most extreme data points without outliers, which are plotted individually (red plus).

To investigate the role of *R. microphyllum*’s tail as a tactile sensor, bats participated in two behavioral experiments. In the first experiment, six bats were released at the bottom of a vertical platform tiled with obstacles (henceforward the maze, Figure 1) with either intact tails (sham trials) or after their tail was anesthetized with lidocaine injections (anesthesia trials, see STAR Methods). The bats’ movement (in all conditions) was characterized by alternating between periods of movement and periods of pausing when encountering an obstacle (Figure S1). In order to test whether the lack of tactile sensation of the tail would affect the bats’ ability to avoid obstacles when moving backwards, we compared the time it took them to climb up and the rate of tail wagging under the two conditions (Video S1). We also used two maze complexity levels: (1) a simple maze with 15 obstacles and (2) a complex maze with 26 obstacles (Figure 1A). The bats solved the simple maze significantly faster than the complex one independently of tail manipulation (generalized linear mixed effect model [GLMM], *p* < 0.0001, *n* = 221 trials, Tables 1 and 2 and see STAR Methods), reaching the top on average 5 s faster (18.7 ± 1.6 vs. 23.7 ± 1.9 s, [mean ± SD]) in the simple and the complex conditions respectively. We averaged across both tail conditions because although the treatment had an effect on performance, the difference between the groups regarding complexity was the same, see Table 2 for specific results). We note that, although bats continuously climbed backwards most of the time, they sometimes stopped to scan an obstacle with their tail, seemingly searching for its perimeter (Video S1). The sensory block treatment also significantly affected the bats’ maze-solving performance under both the simple and complex setups. The sensory block (anesthesia trials) resulted in an increase of ∼2.5 s on average (equivalent of ∼10%) in solving both the simple and the complex maze tasks (GLMM, *p* < 0.0001, *n* = 221, Tables 1 and 2). Additionally, there was an increase of ∼0.15 Hz in the tail wagging rate in the complex maze (GLMM, *p* = 0.03, *n* = 158, Tables 1 and 2; Figure 1C 1–2) and a decrease of ∼1 Hz (∼20%) in rate under sensory block (Table 1; GLMM, *p* < 0.0001, *n* = 158).

**Table 1:** GLMM results of the first experiment

Response	Fixed effect	P - value	Estimate	AIC
Time to climb	Maze complexity	<0.0001	−4.9	1154
	Treatment	<0.0001	2.3	
Tail wagging rate	Maze complexity	0.03	−0.1	107
	Treatment	<0.0001	−0.95	
Movement ratio: Complex maze	Treatment	0.004	0.06	−129.6
Movement ratio: Simple maze	Treatment	0.85	−0.004	−117

Results are shown for the GLMM models: (1) time to climb ∼1 + complexity + treatment + (1 | trial) + (1 | bat); (2) tail wagging rate ∼1 + complexity + treatment + (1 | trial) + (1 | bat); (3) movement ratio ∼1 + treatment + (1 | trial) + (1 | bat).

**Table 2:** Average values of the different experimental conditions

Parameter	Complexity/treatment	Sham	Anesthesia
Time to climb (s)	Simple	17.6 ± 2.5	19.8 ± 2.3
	Complex	22.3 ± 2.7	25 ± 2.2
Tail wagging rate (Hz)	Simple	5.56 ± 0.1	4.46 ± 0.15
	Complex	5.59 ± 0.2	4.7 ± 0.4

Means and standard deviations for all bats are shown for the two parameters: time to climb the maze and tail wagging rate.

Video S1. Obstacle maze from Experiment 1: obstacle avoidance task An example of one individual bat climbing up the obstacle maze, with and without treatment (anesthesia of the tail). A tracking system was used to track markers on the bat’s tail and demonstrates the use of the tail to aid navigating through obstacles, related to Figure 1

To examine if an intact tail improved bats’ ability to negotiate obstacles, we computed the ratio between movements sideways and movements upwards in the direction of the goal, where a larger ratio means less efficient goal-directed movement. Indeed, bats with intact sensing tails performed better in directly reaching the goal in the complex maze (ratio of 1.13 ± 0.04 in anesthesia trials vs. 1.06 ± 0.2 in sham trials, mean ± SD) with a ∼10% less sideways movement than anesthesized bats in the complex maze (GLMM, *p* = 0.004, *n* = 62, for the complex maze, Figure S2; Table 1). There was no difference in movement ratio between the treatment groups in the simple maze (GLMM, *p* = 0.8, *n* = 60, for the simple maze and *p* = 0.1 for the null model that had the best fit, see Tables 1 and S2 and STAR Methods).

### Experiment 2: Texture discrimination

**Figure 2:**
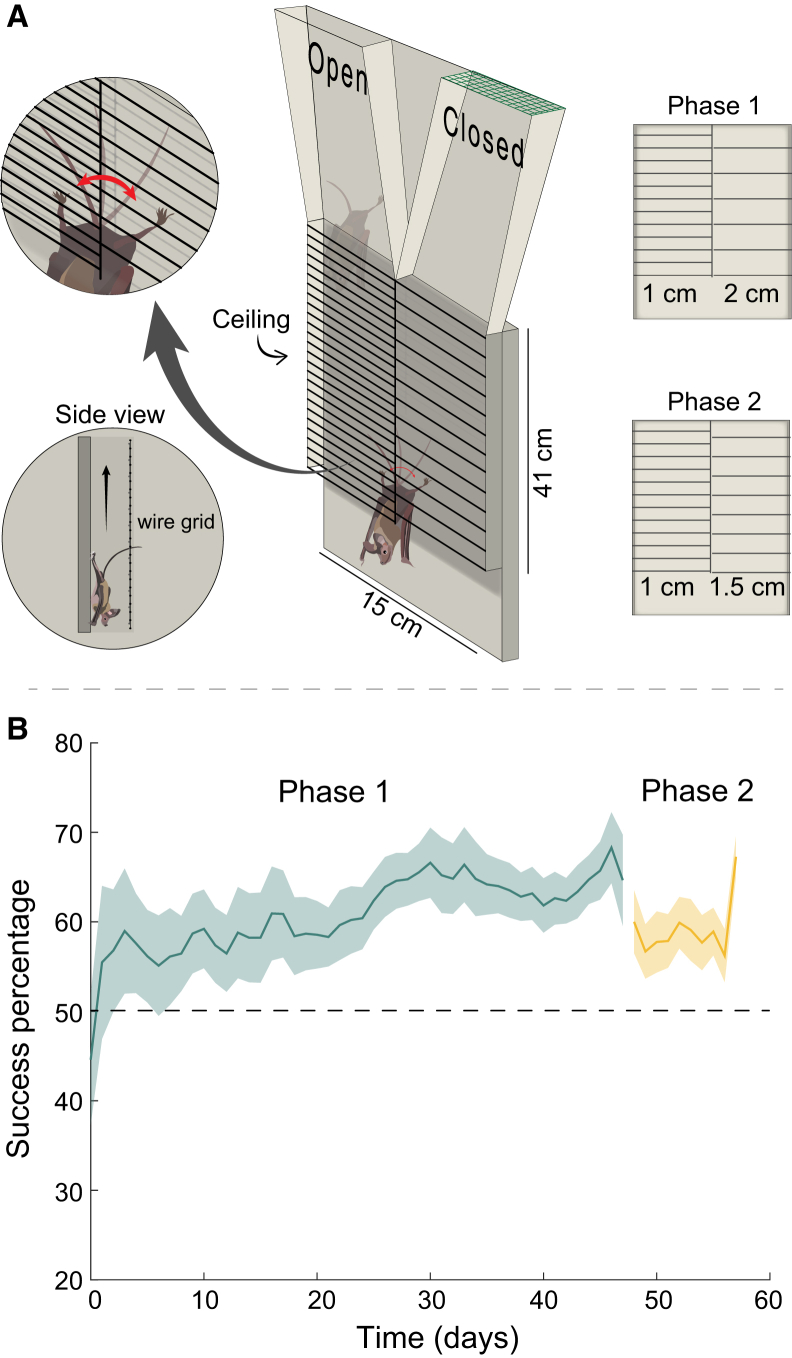
Bats use their tail to distinguish between textures (A) Set-up of the grid Y-mase experiment. The bats had to crawl upwards under wooden bars with different spacing on the right and left sides. Each side led to an open or closed arm that reached the bats’ roosting box. In the first phase of the experiment the spacing was either 1 cm, leading to the open side or 2 cm, leading to the closed side. In the second phase, the 2 cm spacing was changed to 1.5 cm. (B) The success rate over time in the first phase (green) and second phase (yellow). Lines represent the average success percentage per day (*n* = 5 bats) and the shaded areas represent the standard error. The dashed black line represents chance level of 50%.

In a second experiment, we examined tactile texture identification. Five bats were individually released at the base of a vertical Y-shaped maze with two levels of wood grating on the ceiling (see STAR Methods). They were trained to discriminate between a 1 cm grid, that led to an opening of their roosting box (the rewarding side) and a 2 cm grid that led to a closed section of the roosting box (Figure 2 and Video S2). Bats began the first phase of the experiment at chance level, with an average success rate of 46 ± 16% on the first two days of training (binomial test *p* < 0.04 for two bats that significantly differed from 50% chance but were below 50%: 30.8% and 30.8%, and *p* > 0.2 for the other three bats that did not significantly differ from 50% chance: 63.6%, 43.75%, and 61.5%). Over a 48-day training period (phase 1), the bats learned to attribute the 1 cm grating to reward (entering the roosting box), with four of the five bats selecting the rewarding side significantly above chance level during the last 10 days of training (binomial test *p* < 0.03 for four bats while one bat remained at chance level, *p* = 0.08). Next, the grid-width of the non-rewarding side was changed from 2 cm to 1.5 cm (phase 2), to test whether the bats could generalize their learning and to examine whether they could distinguish a 1 cm from a 1.5 cm grating. Three of the four bats (that learned the task) immediately selected the rewarding side significantly above chance (binomial test *p* < 0.05 for three bats).

Video S2. Y-maze from Experiment 2: distinguishing between textures An example of one individual bat climbing up the texture discrimination Y-maze, using its tail to sense the grid on both sides. After reaching the top the bat climbs up one of the arms into the roost, related to Figure 2

Video S3. Anesthesia of the bat’s tail An example of one individual bat’s response to a needle prick in the tail before and after the administration of anesthesia with lidocaine, related to Methods

## Discussion

Animals rely on their senses to navigate their environment, often switching between modalities or combining information from several modalities based on the task.^32^^,^^33^^,^^34^^,^^35^^,^^36^ Some of the best studied modalities include vision,^37^^,^^38^ magnetic sensing,^39^ olfaction,^40^ echolocation,^41^^,^^42^^,^^43^ touch,^44^ and more. The great majority of these sensors are frontal, and located on the head, although some sensors (such as fishes’ side-line) are stretched along the body. The mammalian tail is an elongation of the body used for various tasks including tactile sensing^9^ but not much is known about the sensory use of the tail. In this study, we show that the greater mouse-tailed bat (*Rhinopoma microphyllum*) uses its long, free tail to sense objects and avoid obstacles, as well as discriminate between textures, and to make decisions based on tactile sensation. In the wild, *R. microphyllum* bats roost in small caves crowded with conspecifics and often hang on the walls and crawl backwards (Figure S3), for example when a potential predator enters the cave. Their tails are dynamic and longer than their body, reaching obstacles and surfaces ahead of the legs and body, thus allowing the bat to make predictions about the locations of obstacles and adjust their movement accordingly. Wagging the tail from side to side probably allows *R. microphyllum* bats to assess obstacle or conspecifics’ locations and orientation, and to orient between them as we have also supported by demonstrating that they move more directly toward the goal in a complex environment when their sensing tail is intact. We are not aware of any other bat genus that uses this strategy. Bats in the Tadarida genus (e.g., *Tadarida brasiliensis*) also have a free tail that extends beyond their tail membrane and can be moved voluntarily, but to our knowledge, it remains to be examined whether they use it as a tactile senor.

The tail of *R. microphyllum* performs continuous rhythmic movements that are coupled with the bat’s movement, similar to rat vibrissae. Instead of turning their head or body to use echolocation or vision, these bats only need to wag their tails to detect and avoid obstacles in their way. Moreover, echolocation and vision would not provide useful information when moving backward between obstacles in the dark. We found that blocking the tactile sensors of the tail with anesthesia, reduced both maze-passing duration, and tail-wagging frequency significantly (Figure 1, Video S1). These results indicate that the tactile sensation of the tail and the frequency of probing by tail-wagging are important for navigating in complex environments. The fact that we only find a 10% reduction in the speed of movement should not undermine the result because the bats could also use their bodies to sense and avoid obstacles. It is also possible that our injection did not completely block tail tactile sensing. Rats have evolved a highly sensitive whisker system for orienting efficiently in dark and often narrow environments. By sweeping their whiskers back and forth against objects at frequencies ranging from 5 to 12 Hz,^45^^,^^46^^,^^47^^,^^48^ rats can locate and evaluate objects in their immediate surroundings, even when vision is limited.^48^^,^^49^^,^^50^^,^^51^^,^^52^ *R. microphyllum*’s long tail appears to have a similar function: it improves spatial navigation by rapid probing that increases the sensed range. Rat vibrissae and *R. microphyllum*’s long tail have obvious differences, but the concept of rapidly moving a tactile sensory appendage for spatial navigation is comparable. Similar to rats, the basic tail wagging rate in *R. microphyllum* is 5–6 Hz, but they only slightly increase this rate in a more complex environment, perhaps because the tail is much longer than the whiskers and costly to wave around. The movement of the tail is yet another example of active sensing where sensing is assisted by movement.

It was recently shown that the nectarivorous bat *Glossophaga soricina* rely on their long facial vibrissae to gather positional information relative to a visited flower.^53^ *R. microphyllum* tails are also equipped with several long hairs protruding from their edge which might have special adaptations for tactile sensing.^31^ According to our findings, when tactile sensory cues were inhibited, bats traveled more slowly and required more time to detour obstacles in their surroundings. These findings resemble rats’ behavior when their whiskers were removed^45^^,^^54^ as well as *G. soricina*’s behavior after vibrissae clipping.

We further investigated the role of the tactile properties of the bat’s tail in texture discrimination. Our results show that bats are able to distinguish between different textures when using their tail as a tactile sensor, even between similar gratings of only 0.5 cm difference (1 cm vs. 1.5 cm). The ability to sense and classify texture might come in handy for these bats when deciding whether to hang on a specific wall in their cave. The bats were moreover able to generalize their texture discrimination learning, revealing a high cognitive ability, further highlighting animals’ multisensory nature. Altogether, our results demonstrate that animals are multi-modal and will apply the most suitable sensory system for each specific task.

### Limitations of the study

In this study, we only found a 10% change in speed of climbing backwards after the administration of anesthesia to the bat’s tail in the obstacle maze. Since the bats can use other body parts (e.g., their legs) to sense the environment, diminishing the sensation of the tail alone does not prevent them from successfully moving through the experimental setup. Future studies could look at more detailed tracking of other body parts to provide additional insight into this.

## Resource availability

### Lead contact

Further information and any requests should be directed to and will be fulfilled by the lead contact, Prof. Yossi Yovel (yossiyovel@gmail.com).

### Materials availability

This study did not generate new unique reagents.

### Data and code availability

Data reported in this paper have been deposited at Mendeley Data: https://doi.org/10.17632/kw2thg2xfh.1,^55^ and are publicly available as of the date of publication. This paper does not report original code. Any additional information required to reanalyze the data reported in this paper is available from the lead contact upon request.

## Acknowledgments

We would like to thank Eran Levin for helpful advice regarding the species, Eran Amichai for providing high quality footage from natural caves and Michael Smotherman for consulting about tail movement in Tadarida. We would also like to thank the reviewers for their time and effort in improving this manuscript.

## Author contributions

S.H., O.E., O.D., and Y.Y. conceived and designed the research; S.H. and O.E. collected the data; S.H., O.E., and M.T. analyzed the data; M.T., S.H., and Y.Y. wrote the manuscript; O.E. reviewed and edited the manuscript; All authors read and approved the final manuscript.

## Declaration of interests

The authors declare no competing interests.

## STAR★Methods

### Key resources table

**Table:** 

REAGENT or RESOURCE	SOURCE	IDENTIFIER
**Chemicals, peptides, and recombinant proteins**		

Lidocaine	B. Braun	CAS 137-58-6

**Deposited data**		

Analyzed data	This paper	Mendeley Data https://doi.org/10.17632/kw2thg2xfh.1
Experimental videos	This paper	Mendeley Data https://doi.org/10.17632/kw2thg2xfh.1

**Experimental models: Organisms/strains**		

Six Greater mouse-tailed bats (*Rhinopoma microphyllum*) (3 males and 3 females)	Cave at Northern Israel	Taxonomy ID: 173903

**Software and algorithms**		

MATLAB R2021b	MathWorks	https://www.mathworks.com/downloads/
Cortex-64 6.2.3	Motion - Analysis Corp.	https://www.motionanalysis.com/software/cortex-software/
JMP 17	SAS INSTITUTE	https://www.jmp.com/en_us/software/data-analysis-software.html

**Other**		

Raptor E, 1280 x 1024 pixel, cameras	Motion - Analysis Corp.	Raptor E
Raptor-12, 4096 x 3072 pixel, cameras	Motion - Analysis Corp.	Raptor-12
Basler high speed camera	Basler corp.	acA1300 – 60gmNIR
Ultrasonic speaker Vifa	Avisoft Bioacoustics	https://avisoft.com/playback/vifa/
UltraSoundGate player 216H	Avisoft Bioacoustics	https://avisoft.com/ultrasoundgate/player-216h/

### Experimental model and participant details

Six adult Greater mouse-tailed bats (*Rhinopoma microphyllum*), three males and three females, were captured in a cave at Northern Israel with permission of the Israeli National Park Authority (permit no. 2016/41422). Bats were housed in a wooden box (40 × 40 × 40 cm) in the Zoological Garden at Tel Aviv University. Bats were kept in a reversed light cycle at a temperature of 26°C during their subjective day, and 23°C during their subjective night. The experiment was approved by the institutional IACUC committee number 04-18-041.

### Method details

#### Experiment 1: Obstacle avoidance

##### Experimental setup

In the first experiment, six bats were trained to climb up backwards between two Perspex boards (114 × 24 cm), mimicking a narrowing crack in a cave. The back board was covered with a felt sheet and tiled with obstacles (∼5 × 3 cm^2^ each) that the bats had to detour, thus creating a maze (Figure 1; Table S1). We used two maze arrangements: (1) A simple maze with 15 obstacles; (2) A complex maze with 26 obstacles. These complexity levels are meant to represent possible differences in distance between roost crevices that exist in these bats natural roosting sites (Figure S3). In order to reduce the reliance on spatial memory, the order of the obstacles was changed every experimental day. Social calls were played from the top of the maze to attract the bats upwards, using UltraSoundGate Player 216H (Avisoft Bioacoustics) and an Ultrasonic Omnidirectional Dynamic Vifa Speaker. The bats readily crawled backwards toward the calls so that very little training was required.

Bats were tested under two conditions: (1) treatment – The bats’ tails were locally anesthetized using a 1% Lidocaine injecting to the base of the tail. The injection was performed using a fine insulin needle (BD Micro-Fine plus Demi). The exact dose was calculated per bat according to its weight (and did not exceed 10 mg/kg). The anesthesia took hold instantaneously (in under a minute). This aimed to create a sensory block, while maintaining the tail’s motor abilities intact and prevent an effect on the rest of the body.^56^ A lack of tail response to touch or to a needle prick stimulus was taken as an indicator of sensory block (Video S3). (2) sham – The bats’ tails were injected with saline, to control the effect of the needle perforation. Control bats were able to use their tails normally while climbing. Both the control and sensory-blocked bats were tested first in the simple and later in the complex maze. Three bats began trials under sensory block and three bats began as control and were later alternated. All experiments were conducted in dim light (∼200 millilux), reducing the bats reliance on visual cues. In addition, since the bats were crawling backwards, they could not see the maze obstacles behind them. Moreover, the environment was extremely cluttered in terms of echolocation, with multiple echoes arriving at milliseconds after each emission. It is unlikely that the bats could solve the task based on echoes when moving backwards and only echolocating briefly partially backwards.

##### Tracking and audio recordings

Bats were tracked using a commercial motion - capture system (composed of 16 Raptor E, 1280 x 1024 pixel, cameras and 4 Raptor-12, 4096 x 3072 pixel, cameras, Motion – Analysis Corp.). Bats’ movement was tracked at 200 frames per second, with a spatial accuracy of ∼1 mm.^57^ Six spherical reflectors were placed on the bats using skin bond latex cement (OTSO-BOND Montreal Ostomy Corp). Three 1.6 mm reflectors were placed on the base, middle and tip of the tail; one 2.4 mm reflector was placed on the head and two 6 mm reflectors were placed on the body. Three-dimensional positions of the reflectors placed at the middle of the tail and the bottom of the body were reconstructed using a commercial motion-capture software (Cortex 6.2.3, Motion Analysis, Figure 1A). Further analysis was performed in MATLAB (MATLAB, the Mathworks Inc., Natick, Massachusetts, USA). In addition to the tracking system, a high-speed monochromatic camera, 640 x 480 pixels, recorded the raw video of the movement at 200 fps (Basler, acA1300 – 60gmNIR, Basler corp.). This allowed us to asses tail wagging frequency. In some of the trials a bat-detector was placed at the base of the maze to confirm that the bats were always echolocating while crawling, however, they were echolocating forward and moving backwards so they could not rely on this sensory modality for obstacle avoidance.

The ratio between sideways and upwards movement was computed for each trial based on the movement of the bottom body marker (accumulated sidewise movement/accumulated upwards movement).

#### Experiment 2: Texture discrimination

In a second behavioral experiment, five bats were tested in a two-arm vertical Y-maze (Figure 2; Table S1). The bats were trained to climb backwards, through a corridor (41 cm) that led into the two arms, in order to enter their roosting box (40 × 40 × 40 cm). One arm of the maze led to an opening into the roost while the other was covered in mesh. During the experiments, all other bats were present in the roosting box to allow the tested bat to smell and hear them from both arms. The bats were released one at a time at the base of the maze. The ceiling of the first part of the maze (before the split to two arms) had two different wooden bar-gratings placed side-by-side dividing the corridor’s ceiling into two-halves: one fine - 1 cm grid and one coarse - 2 cm grid (see Figure 2). The fine grid led into the rewarding opening to the roost. The location of the fine grid (on the right or the left) was changed randomly. The experiment had two phases: (1) The first phase - during the first 48 days of the experiment, with the bar gratings as described above. (2) The second phase - during the last 10 days of the experiment, the spaces between the bars leading to the incorrect arm, were changed to 1.5 cm (instead of 2 cm). This phase aimed to examine learning generalization and texture discrimination accuracy.

### Quantification and statistical analysis

#### Experiment 1: Obstacle avoidance

To evaluate the effects of maze complexity and treatment type (e.g., sensory block) in the first experiment, we fitted a Generalized linear mixed effect model (GLMM with fit method of Maximum Pseudo-likelihood). Two response parameters were examined: (1) time to reach the top of the maze (assuming a normal distribution) and (2) rate of tail wagging (frequency; assuming a normal distribution). Maze complexity and treatment (lidocaine vs. sham) were used as fixed effects and bat ID and trial number were set as random effects. After reviewing the Akaike information criterion (AIC) we found that models including the day of trials had a similar fit, and we therefore chose the model with fewer parameters (Table S2). We additionally compared all models to their null model (with only the intercept as an explaining parameter) and found that the null models had a lesser fit with higher AICs.

To estimate the negotiation of obstacles, we performed a GLMM with the ratio of body movement set as the explained parameter, the treatment set as a fixed effect and bat ID and trial number as random effects (assuming a normal distribution, after performing a log transformation of the ratio). We performed two separate GLMMs for each complexity level. We additionally compared these models to the null models (Table S2). Statistical models were performed in MATLAB (R2021b) using the function ‘fitglme’ with an alpha of 0.05. We confirmed that the residuals are approximately normally distributed by examining their distribution and the Q-Q plot in JMP software (SAS INSTITUTE Inc., USA).

#### Experiment 2: Texture discrimination

In the second experiment we used a one-sided binomial test to examine whether the success rate in the Y-maze was significantly higher than 50%. This test was performed for each individual bat. All statistical tests were performed in MATLAB (R2021b).
