# Supplementary material for: Greater mouse-tailed bats use their tail as a tactile sensor when navigating backwards
Source: iScience. 2025 Feb 17;28(3):112014. doi: 10.1016/j.isci.2025.112014 (PMC11927745; doi:10.1016/j.isci.2025.112014)
Supplement: Document S1. Figure S1–S3 and Tables S1, and S2 [file mmc1.pdf]

iScience, Volume *28*

Supplemental information

Greater mouse-tailed bats use their tail as a tactile sensor when navigating backwards

Sahar Hajyahia, Mor Taub, Ofri Eitan, Orit Dashevsky, and Yossi Yovel

Figure S1: Speed of movement through the obstacle maze. The speed of movement (blue line) was computed based on the bottom body marker. The bats' movement (in all conditions) was characterized by alternating between periods of movement and periods of pausing (shaded areas) occurring mostly when encountering an obstacle.

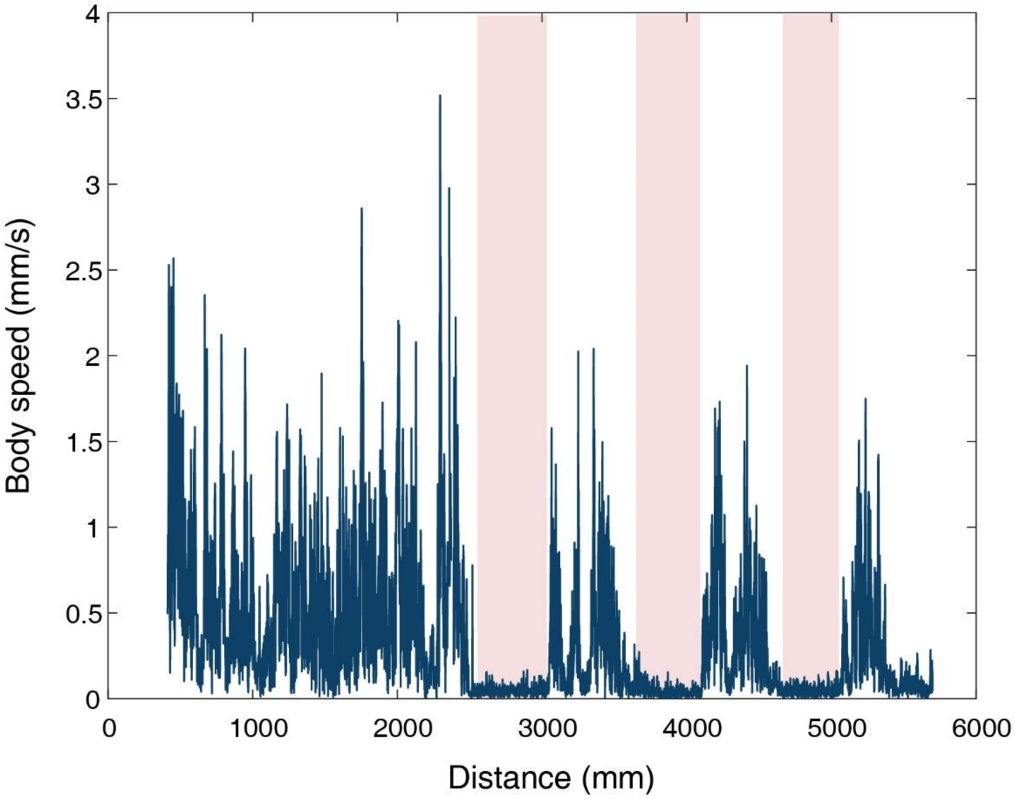

Figure S2: Bats with intact tails move more efficiently in the complex maze. The ratio between sideways movement and upwards movement in the (A) simple and (B) complex obstacle maze under the control (sham) and sensory block treatment (anesthesia). Lines represent the median and lower and upper quartiles. Circles represent individual-trial data points for each condition (mean of each bat, n = 5). The whiskers extend to the most extreme data points without outliers.

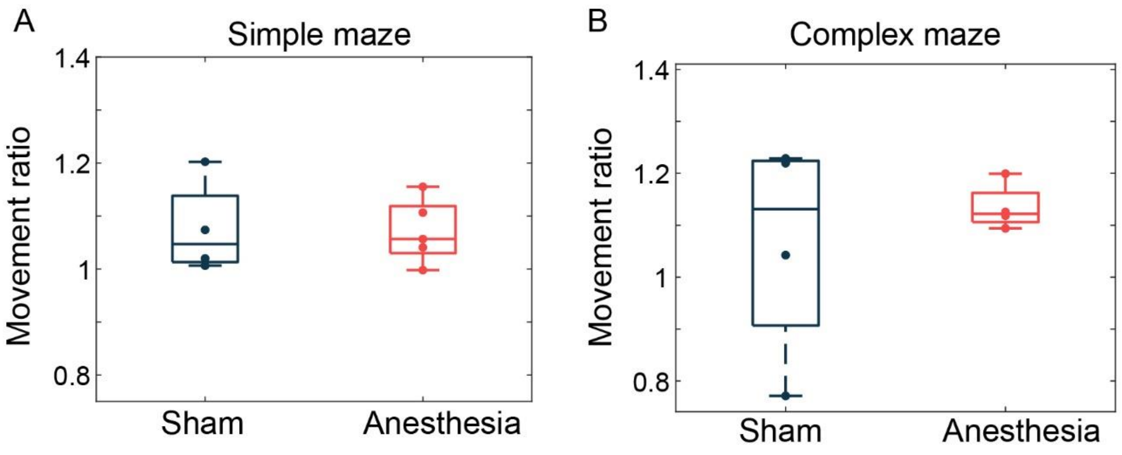

Figure S3: ***Rhinopoma microphyllum's*** **roosts.** Images of roost structures (photos by Eran Amichai) showing different complexity levels of natural and manmade roosts.

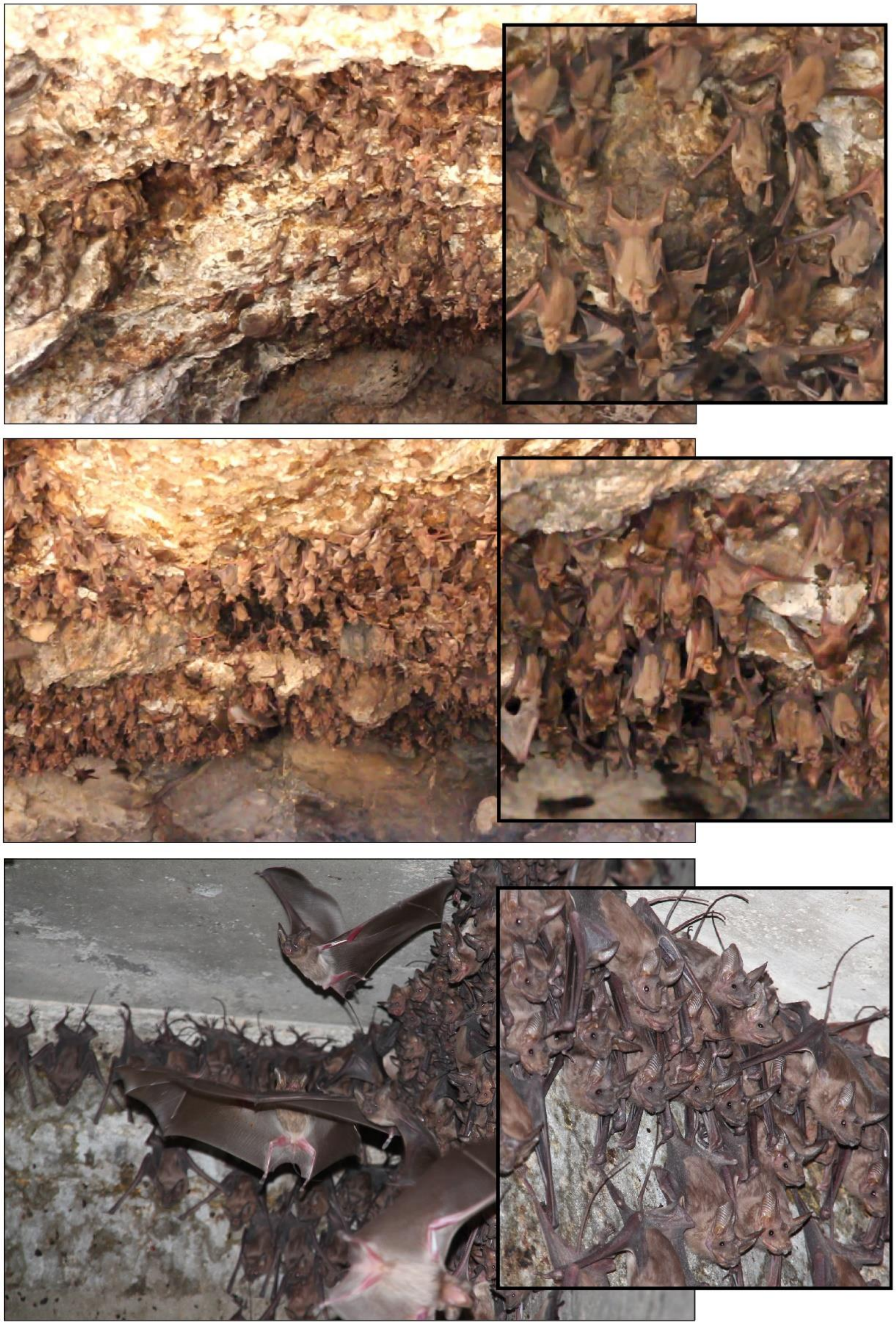

Table S1: Number of trials used for analysis, by individual bats, in the two experiments.

Experiment 1	Simple-sham	Simple-anesthesia	Complex-sham	Complex-anesthesia
Bat 1	10	10	12	14
Bat 2	9	10	11	10
Bat 3	6	10	11	8
Bat 4	10	9	9	9
Bat 5	8	9	5	7
Bat 6	9	10	8	7
**Experiment 2**	**Phase 1-first 2 days (spacing 2)**	**Phase 1-last 10 days (spacing 2)**	**Phase 2 (spacing 1.5)**	
Bat 1	16	131	172	
Bat 2	26	152	183	
Bat 3	11	139	177	
Bat 4	13	137	177	
Bat 6	26	128	178	

Table S2: Additional statistical models. These models had a lesser fit with a higher AIC and therefore not used. Null models are shown for all response parameters.

Response	Effect	P-value	Estimate	AIC
**' Time to finish maze ~ 1 + complexity + treatment + (1|trial) + (1|day) + (1|bat)'**				
**Time to finish maze**	Complexity - low<br>Treatment -anesthesia	<.0001<br><.0001	-5<br>2.3	1154
**' Tail wagging frequency ~ 1 + complexity + treatment + (1|trial) + (1|day) + (1|bat)'**				
**Tail wagging frequency**	Complexity - low<br>Treatment -anesthesia	0.1<br><.0001	-0.1<br>-1	108
**Null models**				
**Time to finish maze**	Intercept	<.0001	21	1301.5
**Tail wagging frequency**	Intercept	<.0001	5	287
**Movement ratio: complex**	Intercept	0.006	0.03	-125
**Movement ratio: simple**	Intercept	0.12	0.02	-123
